# Artificial microRNA-mediated resistance against the monopartite begomovirus *Cotton leaf curl Burewala virus*

**DOI:** 10.1186/1743-422X-10-231

**Published:** 2013-07-12

**Authors:** Irfan Ali, Imran Amin, Rob W Briddon, Shahid Mansoor

**Affiliations:** 1 Agricultural Biotechnology Division, National Institute for Biotechnology and Genetic Engineering, PO Box 577, Jhang Road, Faisalabad, Pakistan

## Abstract

**Background:**

Cotton leaf curl disease, caused by single-stranded DNA viruses of the genus *Begomovirus* (family *Geminiviridae*), is a major constraint to cotton cultivation across Pakistan and north-western India. At this time only cotton varieties with moderate tolerance are available to counter the disease. microRNAs (miRNAs) are a class of endogenous small RNA molecules that play an important role in plant development, signal transduction, and response to biotic and a biotic stress. Studies have shown that miRNAs can be engineered to alter their target specificity. Such artificial miRNAs (amiRNAs) have been shown to provide resistance against plant-infecting viruses.

**Results:**

Two amiRNA constructs, based on the sequence of cotton miRNA169a, were produced containing 21 nt of the V2 gene sequence of *Cotton leaf curl Burewala virus* (CLCuBuV) and transformed into *Nicotiana benthamiana*. The first amiRNA construct (P1C) maintained the miR169a sequence with the exception of the replaced 21 nt whereas in the second (P1D) the sequence of the miRNA169a backbone was altered to restore some of the hydrogen bonding of the mature miRNA duplex. P1C transgenic plants showed good resistance when challenge with CLCuBV; plants being asymptomatic with low viral DNA levels. The resistance to heterologous viruses was lower and correlated with the numbers of sequence mismatches between the amiRNA and the V2 gene sequence. P1D plants showed overall poorer resistance to challenge with all viruses tested.

**Conclusions:**

The results show that the amiRNA approach can deliver efficient resistance in plants against a monopartite begomoviruses and that this has the potential to be broad-spectrum, providing protection from a number of viruses. Additionally the findings indicate that the levels of resistance depend upon the levels of complementarity between the amiRNA and the target sequence and the sequence of the miRNA backbone, consistent with earlier studies.

## Background

RNA interference (RNAi; also known as RNA silencing) is a general phenomenon in eukaryotes that plays diverse roles including defense against pathogens. The key features of RNAi include the production of 21–25 nt small RNAs (sRNA) by enzymes known as Dicers [1] and the formation of RNA-induced silencing complexes (RISCs) which contain Argonaute (Ago) proteins that directly carry out gene silencing at the transcriptional or post-transcriptional levels [2-5]. There are two major classes of sRNAs involved in RNAi, small interfering RNAs (siRNAs) and microRNAs (miRNAs). siRNAs result from the action of dicers on large double-stranded RNAs which have diverse origins. miRNAs are transcribed from their own genes or from introns and from fold-back structures with regions that are double stranded. In plants these primary-miRNAs are processed by the action of dicers into the precursor-miRNA (pre-miRNA) and then miRNA:miRNA* duplexes in the nucleus before being exported for incorporation into RISCs (miRNA* indicating the strand not incorporated into RISC).

Cotton leaf curl disease (CLCuD) is a major constraint to cotton production across Pakistan and north-western India. The disease is caused by monopartite begomoviruses, the most important of which at this time is *Cotton leaf curl Burewala virus* (CLCuBuV), in association with a specific satellite [6]. Viruses of the genus *Begomovirus* (family *Geminiviridae*) have circular single-stranded DNA genomes and are transmitted by the whitefly *Bemisia tabaci*[7]. Although in the Old World a small number of begomoviruses have been identified, with genomes consisting of two ssDNA circles (known as DNA A and DNA B) the majority, including CLCuBuV [8], have genomes consisting of a single component which is a homolog of the DNA A of bipartite viruses. The genomes of monopartite (and DNA A components of bipartite) begomoviruses encode genes in both orientations. The virion-sense strand encodes the coat protein and the V2 gene whereas genes encoded on the complementary-sense strand are involved in viral DNA replication, regulating host and viral gene expression and overcoming host defences [7,8].

Following the demonstration by Vaucheret *et al*. [9] that changes in the sequences of mature miRNAs does not affect their biogenesis and the use of modified miRNAs (referred to as artificial-miRNA [amiRNAs]) to target viruses by Niu *et al*. [10] several studies have been successful in generating resistance to phytopathogenic viruses using amiRNA. Most recently this approach has been successfully applied to a bipartite begomovirus [11]. This has the advantage over approaches that rely on the production of siRNAs, which usually involve quite long virus derived sequences, that only short sequences are required, thus reducing the chances of recombination between the transgenic sequence and the infecting virus. Moreover, use of a 21 nt sequence derived from a begomovirus may avoid silencing of the transgene by the invading virus, as has been reported previously [12]. Here the amiRNA approach has for the first time been investigated as a means of generating resistance to a monopartite begomovirus.

## Results

### Production and analysis of transgenic *N. benthamiana* plants harbouring amiRNA

**Figure 1:**
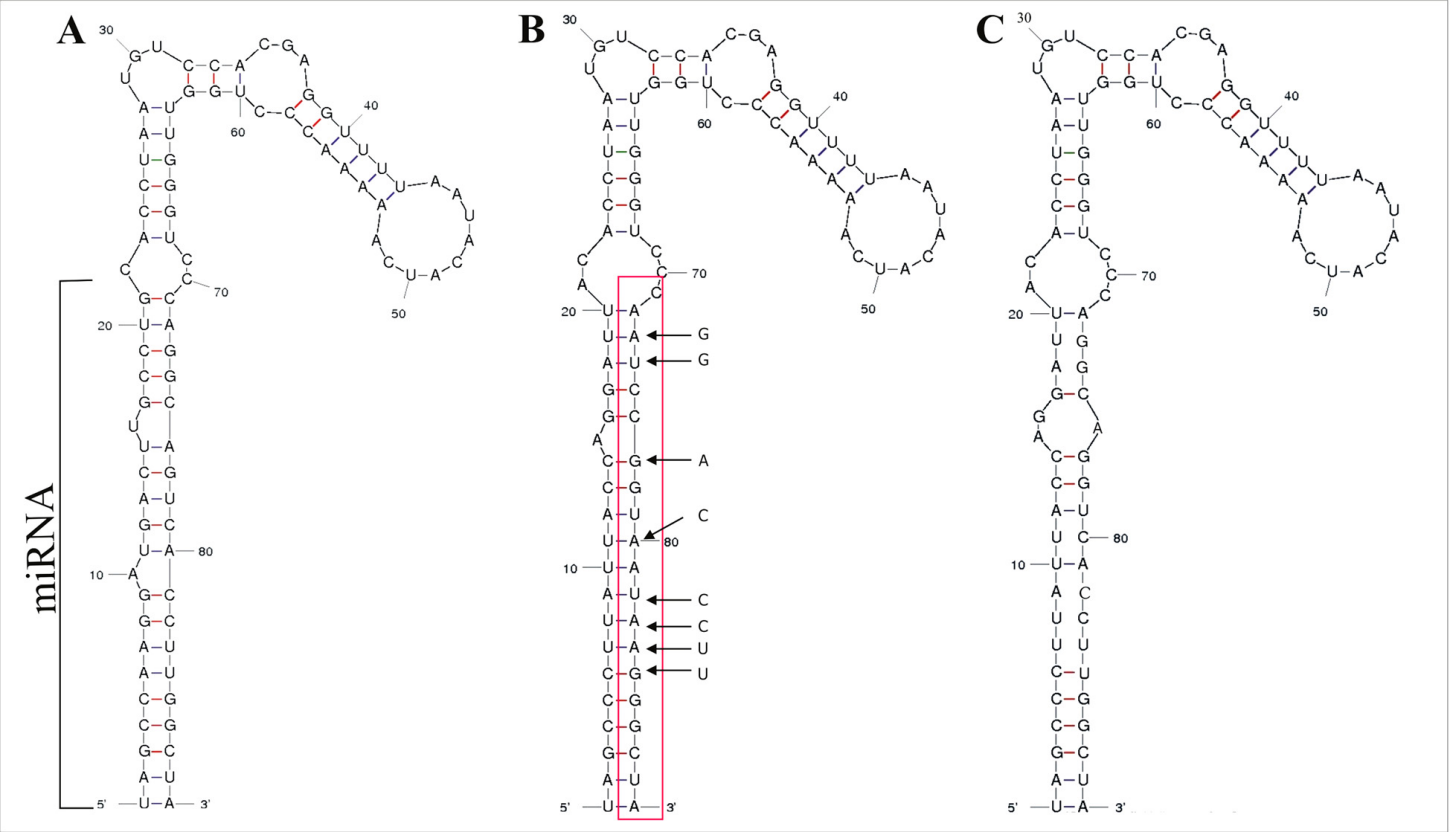
**Predicted secondary structures of pre-miRNAs.** The predicted structures of pre-miRNA169a **(A)**, pre-amiRNA P1D **(B)** and pre-amiRNA P1C **(C)** are shown. For pre-miRNA169a the sequence which forms the mature miRNA is indicated. For the amiRNA this will be the introduced sequence of CLCuBuV. The region of sequence into which changes were introduced into pre-amiRNA P1D to maintain a structure similar to pre-miRNA169a is highlighted by a red box. The nucleotide replaced in the miRNA* sequence are shown by arrows.

The amiRNA produced were based upon the sequence of the pre-miRNA169a (Figure 1A; [13]). For construct P1C the 21 nt of pre-miRNA169a that form the mature miRNA were replaced with 21 nt of the sequence of the V2 gene of CLCuBuV without any further changes (Figure 1C). For construct P1D the same change as in P1C was made but additionally sequence changes were introduced into the sequence of the backbone of pre-miRNA169a (in the sequence that forms the passenger strand [miRNA*] of the mature miRNA duplex) to restore some of the hydrogen bonding in the predicted structure of the pre-miRNA lost by introducing the CLCuBuV sequences (Figure 1B). The introduced sequences are near the center of the V2 gene (coordinates 265–285 of CLCuBuV; EMBL Accession No. AM421522) spanning the area encoding amino acids 46–51 of the predicted 118 amino acid V2 protein.

#### The amiRNA constructs were transformed into *N. benthamiana* by *Agrobacterium*-mediated transformation

A total of 13 Kanamycin resistant primary transformed *N. benthamiana* plants were obtained from the transformation (7 transformed with the P1C construct and 6 with P1D). However, PCR analysis for the presence of the amiRNA indicated that only 3 P1C and 4 P1D plants contained the transgene. These were progressed to the T2 generation by self-pollination and selection of resulting seed on Kanamycin. In the T3 generation a single line for each construct that did not show segregation was selected for virus inoculation. Southern blot analysis of these plants by digestion of extracted DNA with a restriction enzyme that cuts once within the transgene and probing with an *NptII* gene fragment yielded single bands, suggestive of single integration sites for each line (results no shown).

### Challenge inoculation with CLCuBuV

**Figure 2:**
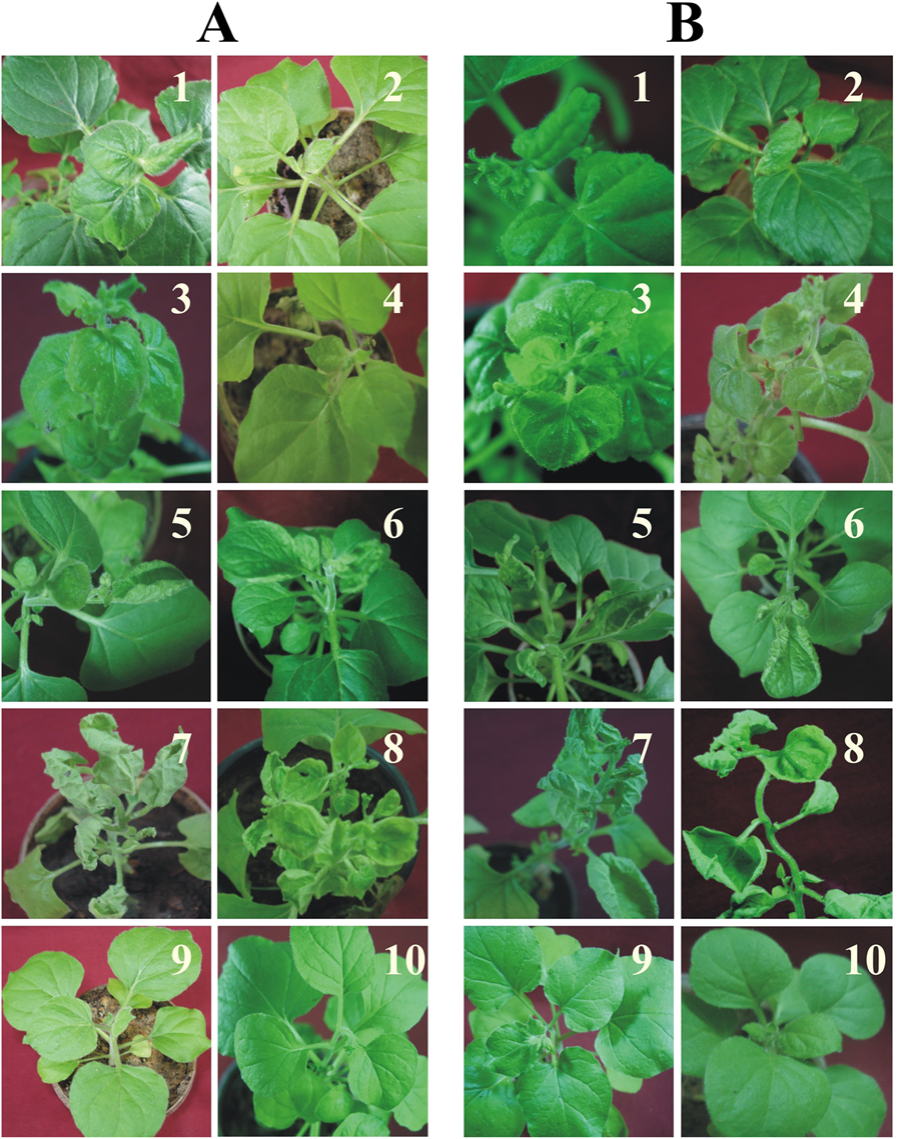
**Symptoms exhibited by transgenic *N. benthamiana *plants following inoculation with begomoviruses.** In each case transgenic plants harbouring the P1C **(A)** or P1D **(B)** constructs were inoculated with CLCuBuV (panel 2), CLCuKoV (panel 4), PedLCuV (panel 6) or ToLCNDV (panel 8). Non-transgenic plants inoculated with CLCuBuV (panel 1), CLCuKoV (panel 3), PedLCuV (panel 5) or ToLCNDV (panel 7) are shown for comparison. Panels 9 and 10 show non-inoculated, non-transgenic plants and mock-inoculated transgenic plants, respectively. Photographs were taken at 28 dpi.

The first symptoms of infection, mild downward curling of newly developing leaves, were visible on non-transgenic *N. benthamiana* plants inoculated with CLCuBuV at 15 days post inoculation (dpi). By 28 dpi all non-transgenic *N. benthamiana* plants showed symptoms of infection consisting of downward leaf curling and some crumpling of newly developing leaves (Figure 2A, panel 1).

**Figure 3:**
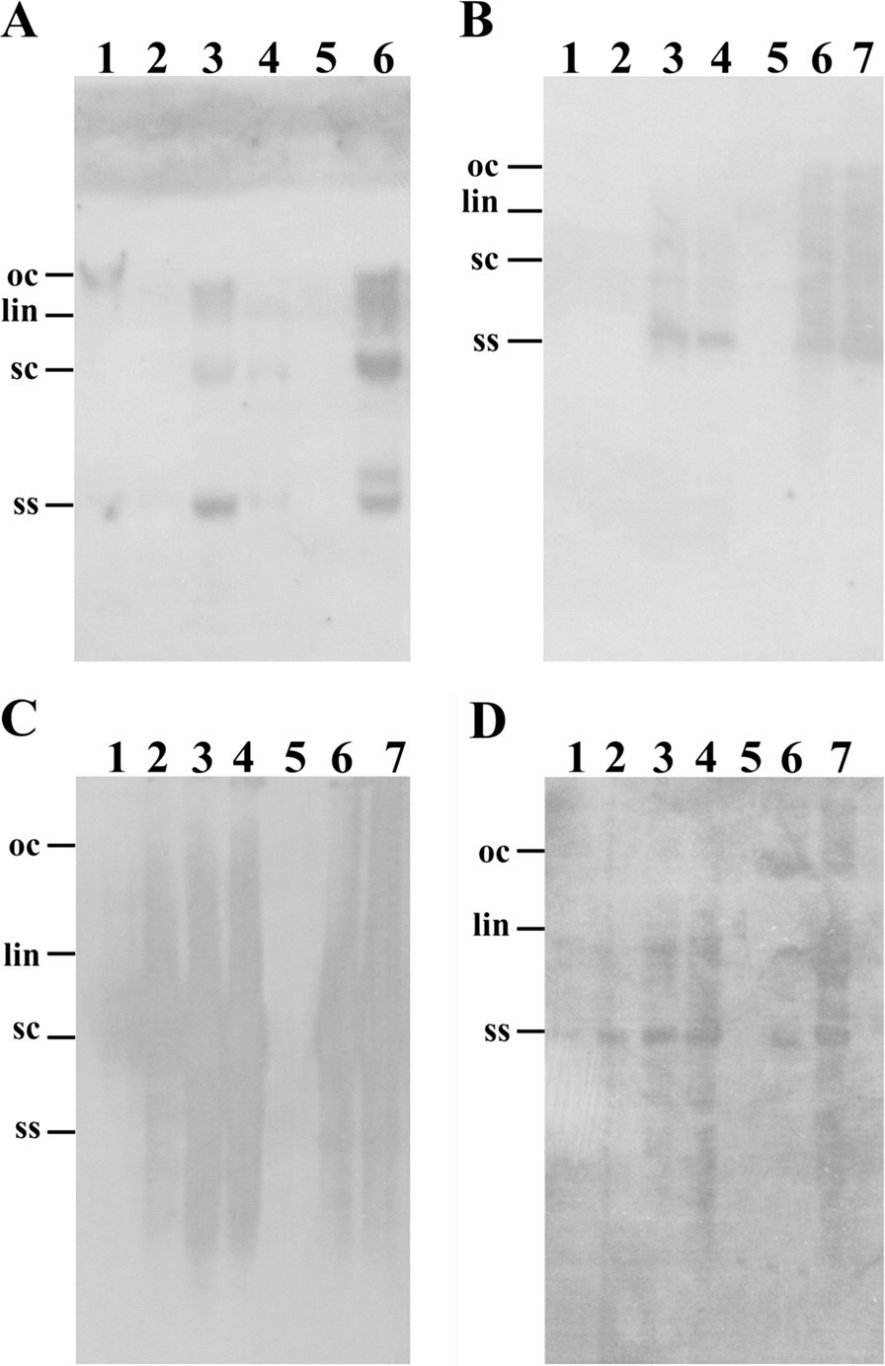
**Southern blot detection of begomoviruses in inoculated *N. benthamiana *plants.** Blots were probed for the presence of CLCuBuV **(A)**, CLCuKoV **(B)**, PedLCuV **(C)** or ToLCNDV **(D)**. For each blot DNA samples were extracted from inoculated transgenic plant harbouring the P1C (lanes 1 and 2) or P1D (lanes 3 and 4) constructs or inoculated non-transgenic plants (lanes 6 and 7; note DNA from only a single plant was used in panel **A**). DNA extracted from a non-inoculated transgenic plant was run in lane 5. Approx. 10 μg of total DNA was loaded in each case. The positions of viral single-stranded (ss), super-coiled (sc), linear (lin) and open-circular (oc) DNA forms are indicated.

For CLCuBuV inoculated transgenic *N. benthamiana* plants harbouring P1C a small number of plants (3 out of 20 plants inoculated) showed very mild symptoms at 15 dpi (Figure 2A, panel 2). However, these symptoms did not develop into the severe symptoms shown by non-transgenic plants, with all subsequently developing leaves showing no symptoms. PCR analysis showed the majority of plants (19 out of 20) contain viral DNA (Table 1). Southern blot analysis of DNA extracted from inoculated plants showed that plants that ex hibited the initial mild symptoms contained slightly more viral DNA than plants that remained asymptomatic (Figure 3, panel A, lanes 1 and 2). However, the transgenic plants all contained significantly lower viral DNA levels than infected, non-transgenic plants (Figure 3, panel A, lane 6).

**Table 1:** **Infectivity of CLCuBuV in transgenic *N. benthamiana *plants harbouring amiRNA**

**Plant**	**Infectivity**				**Diagnostics**		
	**(plants symptomatic/plants inoculated)**						
	**Exp. 1**		**Exp.2**		**PCR**^**£**^		**Southern**^**$**^
	**15dpi**	**28dpi**	**15dpi**	**28dpi**	**Exp. 1**	**Exp.2**	
*N. b*	8/10	10/10	10/10	10/10	9/10	10/10	++++
P1C	2/10	0/10	1/10	0/10	10/10	10/10	+
P1D	3/10	8/10	2/10	10/10	10/10	10/10	+++
N. b*	0/10	0/10	0/10	0/10	0/10	0/10	ND
*N. b*^*#*^	0/5	0/5	0/5	0/5	0/5	0/5	−

* Non-transgenic *N. benthamiana* plants inoculated with *Agrobacterium* cultures harbouring pGreen0029.

^*#*^ Non-inoculated plants.

^£^ CLCuBuV was detected in nucleic acids extracted from plants by PCR using primers IRVF and IRVR (Table 5).

^$^ Southern hybridization results are given as strong hybridization (++++), through weak hybridization (+), to no hybridization detected (−). Some plants were not examined for the presence of virus by hybridization (ND).

P1D transgenic *N. benthamiana* plants inoculated with CLCuBuV showed the first mild symptoms of infection at 15 dpi in a small number of plants (5 out of 20 plants inoculated). However, these plants did not continue to show severe symptoms with all newly developing leaves showing very mild leaf curling (18/20). All other plants remained symptomless throughout (Figure 2B, panel 2). Southern blot hybridization showed the presence of viral DNA at levels below those detected in infected, non-transgenic plants (Table 1).

### Challenge inoculation with *Cotton leaf curl Kokhran virus*

For non-transgenic *N. benthamiana* plants inoculated with *Cotton leaf curl Kokhran virus* (CLCuKoV) the first symptoms of infection, consisting of a mild downward leaf curling of the edges of newly developing leaves, were observed at 21 dpi. All non-transgenic plants developed severe downward leaf curling symptoms in newly developing leaves by 28 dpi (Figure 2A, panel 3).

None of the transgenic *N. benthamiana* plants harbouring the P1C construct developed symptoms following inoculation with CLCuKoV (Figure 2A, panel 4). However, diagnostic PCR with primers CLCKV2F and CLCKV2R showed the presence of viral DNA in young, newly developing leaves even though no viral DNA could be detected by Southern blot hybridization (Figure 3b; Table 2). This suggests that viral DNA levels were below the detection threshold for hybridization.

**Table 2:** **Infectivity of CLCuKoV in transgenic *N. benthamiana *plants harbouring amiRNA**

**Plant**	**Infectivity**				**Diagnostics**		
	**(plants symptomatic/plants inoculated)**						
	**Exp. 1**		**Exp.2**		**PCR**^**£**^		**Southern**^**$**^
	**15dpi**	**28dpi**	**15dpi**	**28dpi**	**Exp. 1**	**Exp.2**	
*N. b*	0/10	10/10	1/10	10/10	10/10	10/10	++++
P1C	0/10	1/10	0/10	0/10	10/10	10/10	+
P1D	0/10	8/10	0/10	10/10	10/10	10/10	++
N. b*	0/10	0/10	0/10	0/10	0/10	0/10	ND
*N. b*^*#*^	0/5	0/5	0/5	0/5	0/5	0/5	−

* Non-transgenic *N. benthamiana* plants inoculated with *Agrobacterium* cultures harbouring pGreen0029.

^*#*^ Non-inoculated plants.

^£^ CLCuKoV was detected in nucleic acids extracted from plants by PCR using primers CLCKV2F and CLCKV2R (Table 5).

^$^ Southern hybridization results are given as strong hybridization (++++), through weak hybridization (+), to no hybridization detected (−). Some plants were not examined for the presence of virus by hybridization (ND).

The first symptoms of infection on transgenic *N. benthamiana* plants harbouring P1D following inoculation with CLCuKoV were visible in a small number of plants at 21 dpi and the majority of plants showed severe symptoms of infection by 28 dpi (Figure 2B, panel 4; Table 2). Southern blot analysis showed the presence of significant amounts of viral DNA in P1D transgenic plants, although this was lower than the levels of viral DNA detected in infected, non-transgenic plants (Figure 3, panel B).

### Challenge inoculation with *Pedilanthus leaf curl virus*

The first symptoms of infection, a mild upward curling of the edges of newly developing leaves, in non-transgenic *N. benthamiana* plants inoculated with *Pedilanthus leaf curl virus* (PedLCuV) were visible at 15 dpi. By 21 dpi all non-transgenic plants exhibited severe symptoms consisting of upward leaf curling, vein thickening and leaf shortening (Figure 2A, panel 5; Table 3).

**Table 3:** **Infectivity of PedLCuV in transgenic *N. benthamiana *plants harbouring amiRNA**

**Plant**	**Infectivity**				**Diagnostics**		
	**(plants symptomatic/plants inoculated)**						
	**Exp. 1**		**Exp.2**		**PCR**^**£**^		**Southern**^**$**^
	**15dpi**	**28dpi**	**15dpi**	**28dpi**	**Exp. 1**	**Exp.2**	
*N. b*	10/10	10/10	10/10	10/10	10/10	10/10	++++
P1C	3/10	10/10	1/10	10/10	10/10	10/10	+++
P1D	8/10	10/10	10/10	10/10	10/10	10/10	++++
N. b*	0/10	0/10	0/10	0/10	0/10	0/10	ND
*N. b*^*#*^	0/5	0/5	0/5	0/5	0/5	0/5	−

* Non-transgenic *N. benthamiana* plants inoculated with *Agrobacterium* cultures harbouring pGreen0029.

^*#*^ Non-inoculated plants.

^£^ PedLCuV was detected in nucleic acids extracted from plants by PCR using primers PedLCVV2F and PedLCuVV2R (Table 5).

^$^ Southern hybridization results are given as strong hybridization (++++), through weak hybridization (+), to no hybridization detected (−). Some plants were not examined for the presence of virus by hybridization (ND).

Mild symptoms of infection appeared in 3 out 10 inoculated transgenic plants harbouring P1C construct but all the plants showed severe symptoms by 21 dpi (Figure 2A, panel 6). In contrast, for plants transformed with the P1D construct, all inoculated plants showed initial mild symptoms at 15 dpi and severe symptoms by 21 dpi (Figure 2B, panel 6; Table 3). Southern blot hybridization of genomic DNA extracted from systemic leaves showed the accumulation of high levels of viral DNA in non-transgenic and P1D transgenic *N. benthamiana* plants (Figure 3, panel C). However, in transgenic plants harboring P1C possibly slightly less viral DNA accumulated than in non-transgenic plants.

### Challenge inoculation with *Tomato leaf curl New Delhi virus*

Non-transgenic *N. benthamiana* plants inoculated with the bipartite *Tomato leaf curl New Delhi virus* (ToLCNDV) showed the first symptoms of infection, mild leaf curling, at 15 dpi. By 21 dpi all plants were symptomatic showing upward leaf curling, leaf yellowing, vein thickening and a reduction in leaf size (Figure 2A, panel 7).

All transgenic plants inoculated with ToLCNDV behaved like the non-transgenic plants with initial symptoms appearing at 15 dpi and full symptoms in all plants developing by 21 dpi (Figure 2A, panel 8; Figure 2B, panel 8; Table 4). Southern blot analysis of inoculated plants showed the levels of ToLCNDV DNA in transgenic plants harbouring the P1D construct to be approximately equal to the levels of viral DNA detected in non-transgenic plants (Figure 3, panel E). However, for transgenic plants harbouring the P1C construct, the levels of ToLCNDV DNA were possibly marginally lower.

**Table 4:** **Infectivity of ToLCNDV in transgenic *N. benthamiana *plants harbouring amiRNA**

**Plant**	**Infectivity**				**Diagnostics**		
	**(plants symptomatic/plants inoculated)**						
	**Exp. 1**		**Exp.2**		**PCR**^**£**^		**Southern**^**$**^
	**15dpi**	**28dpi**	**15dpi**	**28dpi**	**Exp. 1**	**Exp.2**	
*N. b*	10/10	10/10	9/10	10/10	10/10	10/10	++++
P1C	9/10	10/10	7/10	10/10	10/10	10/10	+++
P1D	10/10	10/10	10/10	10/10	10/10	10/10	++++
N. b*	0/10	0/10	0/10	0/10	0/10	0/10	−
*N. b*^*#*^	0/5	0/5	0/5	0/5	0/5	0/5	−

* Non-transgenic *N. benthamiana* plants inoculated with *Agrobacterium* cultures harbouring pGreen0029.

^*#*^ Non-inoculated plants.

^£^ ToLCNDV was detected in nucleic acids extracted from plants by PCR using primers ToLCNDVV2F and ToLCNDVV2R (Table 5).

^$^ Southern hybridization results are given as strong hybridization (++++), through weak (+) hybridization to no hybridization detected (−).

## Discussion

Since the phenomenon was first identified RNAi has become the strategy of choice in efforts to develop transgenic resistance against viruses in plants. The siRNA strategy has been shown to be widely applicable against phytopathogenic viruses, including geminiviruses [14]. More recently engineered miRNAs have been investigated as a means of obtaining resistance following the demonstration that the targeting sequences pre-miRNAs could be modified [10]. This approach has been shown to effectively deliver resistance against viruses including, most recently, the bipartite begomovirus ToLCNDV [11,15,16]. The results obtained here show for the first time that the amiRNA may also deliver resistance against monopartite begomoviruses.

The P1C amiRNA construct delivered efficient resistance against both CLCuBuV and CLCuKoV. The majority of plants remained symptomless and the few plants that developed mild symptoms initially proceeded to lose those symptoms. Nevertheless, all CLCuBuV inoculated P1C plants were shown to contain viral DNA by PCR but (at least for plants that did not show the initial symptoms) the levels of DNA were below the detection threshold of Southern blot hybridization and significantly lower than infected non-transgenic control plants.

**Figure 4:**
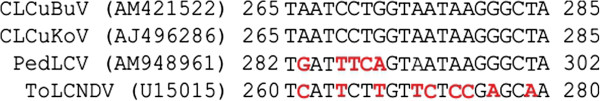
**Alignment of the V2 gene sequences homologous to the amiRNA of the bogomoviruses used.** Shown are the sequences of the V2 genes of *Cotton leaf curl Burewala virus* (CLCuBuV), *Cotton leaf curl Kokhran virus* (CLCuKoV) and *Pedilanthus leaf curl virus* (PedLCuV) as well as the AV2 gene of *Tomato leaf curl New Delhi virus* (ToLCNDV). Nucleotides differing from those of the CLCuBuV sequence are highlighted in red. Note that the sequences of amiRNAs P1C and P1D target (are complimentary to) the CLCuBuV sequence.

Less efficient resistance in P1C plants was evident to PedLCuV and ToLCNDV infections. Plants inoculated with PedLCuV showed delayed symptoms, although ultimately the viral DNA levels were high but lower than in non-transgenic plants. Inoculation of P1C plants with ToLCNDV had no apparent effects on the timing of symptom appearance or symptom severity. Nevertheless in some plants the levels of viral DNA were lower than in infected non-transgenic plants. An alignment of the V2 (AV2 for ToLCNDV) gene sequences homologous to those of the amiRNA of the four begomoviruses is shown in Figure 4. This shows the sequences of the V2 genes of the CLCuBuV and CLCuKoV to be identical in the region targetted by the P1C amiRNA. In contrast the V2 of PedLCV and the AV2 of ToLCNDV show mismatches (5 nucleotides for PedLCuV and 9 for ToLCNDV). The relative levels of resistance detected thus correlates with the levels of sequence identity, consistent with RNAi being homology dependent process [17].

Overall the P1D amiRNA gave much poorer resistance although, with the possible exception of ToLCNDV, some symptom amelioration or reduction in viral DNA levels in plants was detected. This shows that the resistance resulting from amiRNA is influenced by the backbone sequence. This may be due to expression levels of the amiRNA or its interaction with Ago proteins, both of which have been shown to be affected by the sequence of the backbone [14,18].

Mutation of the V2 gene of monopartite begomoviruses leads to viruses that induce infections that are non-symptomatic with very low viral DNA levels [19-21], indicating that the V2 protein is a pathogenicity determinant and possibly involved in virus movement in plants. These features of V2 mutants are thus equivalent to the effects of silencing of the V2 gene using an amiRNA shown here. The product of the AV2 gene of bipartite begomoviruses is not essential for infectivity and mutation of these results in viruses that induce delayed but symptomatically normal infections [22], suggesting that this protein plays in part in, but is not essential for, virus movement; the essential virus-encoded proteins being encoded on the second component (DNA B; [23]). Although it is likely that the poor levels of resistance seen here to ToLCNDV are due to sequence differences with the amiRNA, it is also likely that part of the effect is due to complementation of the silenced AV2 gene functions by the DNA B. In light of this it is somewhat surprising that the amiRNA-mediated resistance targeting virion-sense genes of ToLCNDV reported by Vu *et al*. [11] resulted in either mild transient or no symptoms of infection. Possibly this is a host specific difference; the earlier study having used tomato whereas the study here used the highly permissive host *N. benthamiana.*

Manavella *et al*. [24] have shown that the use of 22 nt miRNA* or miRNA: miRNA* duplexes with asymmetric bulges can trigger transitivity–spread of the silencing outside the targeted sequence by the production of secondary siRNAs. Making use of this phenomenon may be useful in improving miRNA-mediated resistance against geminiviruses with the possibility of inducing TGS and should be investigated in the further studies. Also targeting multiple transcripts with distinct amiRNA may yield improved resistance and reduce the chance of the resistance being broken by sequence changes in the virus.

The inability of the amiRNA to yield immunity, even against the homologous virus, suggests that this approach is unlikely to yield a durable resistance. Although virus levels are low in transgenic plants, the presence of replicating virus would allow mutations in the target sequence to accumulate leading, eventually, to a virus able to overcome the resistance. This is a problem with all homology-based resistance strategies and is particularly significant for the amiRNA approach, where only a short sequence is targeted. There are a number of possible ways of overcoming this problem. Firstly the durability may be improved by designing the amiRNA to sequences that are more essential to the virus than those used here, such as the sequences of active sites of virus encoded proteins which are likely to prove less likely to change by mutation. Another possibility is to target both complementary and virion-sense sequences by multiple amiRNAs. Secondly the amiRNA resistance may be “pyramided” with additional resistances that act by distinct mechanisms, making it less likely that mutation will lead to susceptibility [25].

The results of the study conducted here show that transgenic expression of an engineered amiRNA can efficiently counter infection of a monopartite begomovirus but does not lead to immunity. The ability of the expressed miRNA to deliver resistance against heterologous viruses being depended upon the levels of complementarity between the miRNA and the target is consistent with RNAi being a sequence homology-dependent phenomenon. Additionally, the sequence of the miRNA backbone was shown to influence the levels of resistance obtained.

## Methods

### Production of amiRNA expression constructs

The amiRNA constructs were based on the backbone sequence of the cotton pre-miR169a [13] and were synthesized by Macrogen (Korea). Synthetic amiRNA were transferred to the expression vector pJIT163 under the control of the *Cauliflower mosaic virus* 35S promoter and nos terminator [26] as *Eco*R1 and *Hin*dIII fragments. Resulting expression cassettes were cloned in the binary vector pGreen0029 [27].

### Transformation of *N. benthamiana* and infectivity assays

*N. benthamiana* was transformed by the leaf disc method using *Agrobacterium tumefaciens* GV3101 [28]. *Agro bacterium*-mediated inoculation of plants with CLCuBuV (AM421522; [8]), CLCuKoV (AJ496286; [29]), PedLCuV (AM948961; [30]) and ToLCNDV (DNA A [U15015] and DNA B [U15017]; [31]) was conducted as previously described by infiltration using 5 ml syringes [32].

### Extraction of total nucleic acids from plants and detection of viral DNA

DNA was extracted from plants using the CTAB method [33]. Viral DNA was detected in extracts by PCR-mediated diagnostics using primer pairs IRVF/IRVR for CLCuBuV, CLCKV2F/CLCKV2R for CLCuKoV, PedLCVV2F/ PedLCuVV2R for PedLCuV and ToLCNDVV2F/ToLCNDVV2R for ToLCNDV (Table 5).

**Table 5:** List of oligonucleotides primers used in the study

**Primer**	**Sequence (5′-3′)**	**Virus/ Construct**	**Size of amplicon (bp)**	**Amplicon**
BAF	ACGCGTGCCGTGTTGCTGCCCCATTGTCC	begomovirus	~2800	genome
BAR	ACGCGTATGGGCTGYCGAAGTTSASACG			
BV2F	ATGTGGGATCCACTGTTAAATGAG	CLCuBuV	356	V2 gene
BV2R	TAGGAACATCTGGACTTCTGTAC			
CLCKV2F	GTCGACAAGTATGCGTTTGAAAAATGTG	CLCuKoV	370	V2 gene
CLCKV2R	GGATCCACCTTCACATCCTCTAGGAAC			
IRVF	CGTGGAATTCATGTGGGATCCACTGTT	CLCuBuV	348	V2 gene
IRVR	TTCGTCGACGAACATCTGGACTTCTGTA			
PedLCV2F	ATGTGGGATCCGTTATTGAAC	PedLCuV	357	V2 gene
PedLCV2F	CTAGGAACATCTGGACTTCTG			
TLCNDV2F	GTCGACAAACATGTGGGATCCATTATTGC	ToLCNDV	361	AV2 gene
TLCNDV2R	CCCGGGCTTCTATACATTCTGTAC			
P1CF	GACAGTGGTCCCAAAGATGGA	P1C	273	expression cassette*
P1CR	ATCGGGAATAATGGTCCTAA			
P1DF	GACAGTGGTCCCAAAGATGGA	P1D	275	expression cassette*
P1DR	TAGCCCATATGCCCGGATTG			
35SF	GACAGTGGTCCCAAAGATGGA	pJIT163	150	CaMV 35S promoter
35SR	CAGTGGAGATATCACATCAATCCA			
TLCNDBF	GTCCCGGGTATGTCACGTATCCATCA	ToLCNDV DNA B	550	NSP gene
TLCNDBR	TATCGATTTATCCAATGTAATTAAGAAT			

* These primer pairs amplify from the 3′ end of the CaMV 35S promoter (P1CF and P1DF) to the 3′ end of the amiRNA construct (primers P1CR and P1DR being the reverse complement of the sequence of the amiRNA backbone [amiRNA* sequence]).

Viral DNA was detected by Southern blot hybridization essentially as described previously [34]. DNA was resolved on 1% agarose gels in 0.5 × TAE buffer and transferred to nylon membrane (Hybond N+, GE Healthcare). Blots were hybridized with viral DNA fragments produced by PCR with the primers listed in Table 5 and labeled with DIG using a PCR DIG probe synthesis kit (Roche, Switzerland) according to manufacturer’s protocol.

## Competing interests

The authors declare that they have no competing interests.

## Authors’ contributions

IrA conducted all experiments and wrote the first draft of the manuscript. ImA, SM and RWB edited the paper and coordinated the research work. All authors have read and approved the manuscript.
